# Assessment of Pozzolanic Activity Using Methods Based on the Measurement of Electrical Conductivity of Suspensions of Portland Cement and Pozzolan

**DOI:** 10.3390/ma7117533

**Published:** 2014-11-21

**Authors:** Sergio Velázquez, José M. Monzó, María V. Borrachero, Jordi Payá

**Affiliations:** 1 Facultad de Ingeniería, Universidad Panamericana Campus Guadalajara, Prolongación Calzada Circunvalación Poniente No. 49, 54010 Zapopan, Mexico; 2 Instituto de Ciencia y Tecnología del Hormigón (ICITECH), Universitat Politècnica de València, Camino de Vera s/n, 46071 Valencia, Spain; E-Mails: jmmonzo@cst.upv.es (J.M.M.); vborrachero@cst.upv.es (M.V.B.); jjpaya@cst.upv.es (J.P.)

**Keywords:** spent fluid catalytic cracking catalyst, pozzolanic activity, electrical conductivity measurement, cement suspension

## Abstract

The use of methods based on measuring electrical conductivity to assess pozzolanic activity has recently been used primarily in aqueous suspensions of pozzolan: calcium hydroxide. However, the use of similar methods in suspensions of cement with pozzolans has not been widely studied. This paper proposes a new method for rapid assessment of the pozzolanic activity of mineral admixtures in aqueous cement suspensions. In this study, the conditions for the application of the method were optimized, such as time, temperature, *w*/*c* ratio and dosage procedure. Finally, results are presented from the application of this method for characterizing the pozzolanic activity of the spent catalytic cracking catalyst. These results corroborate as previously reported, namely the high reactivity of this pozzolan obtained by other methods, such as thermogravimetry or evolution of the mechanical strength. In addition, the pozzolanic activity of the catalyst was compared with other pozzolans such as metakaolin and silica fume.

## 1. Introduction

The development of analytical methods that reduce assay time has always been a concern of researchers. The study of the pozzolanic activity of mineral additions has not been the exception. In this sense, one of the proposed methods to this effect has been based on electrical conductivity measurements in aqueous suspensions of pozzolan: calcium hydroxide [1,2,3,4,5,6,7,8,9,10]. In these studies, the direct reaction between the acid reactive components of the pozzolan (SiO_2_, Al_2_O_3_) and the calcium hydroxide is evaluated. However, there are very few references related to electrical conductivity studies of cement suspensions, and even less studies aimed at evaluating the pozzolanic activity of mineral admixtures in the presence of cement. One of these references is drawn from Sintharworn *et al.* [4,5]. They studied pozzolanic activity by measuring the electrical conductivity of suspensions obtained by mixing several pozzolans (silica fume, metakaolin and rice husk ash) using a solution obtained from the filtration of a mix of Portland cement and water. The researchers analyzed the influence of calcium hydroxide concentration and the suspension temperature. However, it is clear that the process studied is the direct reaction between pozzolan and calcium hydroxide dissolved. Lanzón *et al.* [11] studied the influence of lightweight aggregates in mortar performance. These aggregates showed a slight pozzolanic activity, which was studied, on the one hand in aqueous suspensions of calcium hydroxide, and on the other hand in aqueous suspensions of Portland cement. Their experiments for cement suspensions lasted for a week. In the results of the time trial, the lightweight aggregate samples showed a decrease in electrical conductivity due to the pozzolanic reaction. Maximilien *et al.* [12] investigated the reactivity of six cements by measuring the electrical conductivity of aqueous suspensions using no mineral addition. As a result of their studies, three periods are established in the hydration reaction of cement: mixing, induction (dormant) and acceleration. These periods are defined depending on the changes in the values of electrical conductivity of the aqueous suspension of cement. The first period is characterized by an elevation, in a few minutes, of electrical conductivity. This elevation is due to the rapid dissolution of the cement components, causing supersaturation. In the second period (induction), a slow rise in electrical conductivity continues, where reactions proceed slowly. A supersaturation of portlandite occurs, reaching a maximum in electrical conductivity, which defines the end of this period. In the final period (acceleration), the reactions are accelerated due to the precipitation of portlandite, giving as a result a sudden decrease in electrical conductivity, and the dissolved ions are consumed by precipitation. Table 1 show a summary of the main characteristics of these previous methods of electrical conductivity measurements in aqueous suspensions where Portland cement is involved.

Regarding the spent catalytic cracking catalyst (FCC), a previous work [13] has reported the study of pozzolanic activity in aqueous suspensions of FCC: calcium hydroxide. This study demonstrated the high reactivity of this material, comparing their behavior with metakaolin. The influence of the temperature of the suspension was reported along with the influence of the pozzolan/calcium hydroxide ratio. To evaluate the pozzolanic activity of the catalyst residue (FCC) in cement suspensions, several experimental conditions must be established in order to implement the method of electrical conductivity previously used in aqueous suspensions of pozzolan/calcium hydroxide [13], such as:

- Water/cement ratio of the suspension. Maximilien *et al.* [12] used a ratio of 4:1, which is very low with respect to the water/solid ratio that was used in the lime suspensions [13], where the minimum ratio used was 25:1.
- For the process of adding the pozzolan, there were two options. One was adding the pozzolan simultaneously with cement, which would give us information about the interaction between the pozzolan and calcium hydroxide consumption, which is generated by cement hydration. However, the dissolution of the cement salts could mask the pozzolanic effect, which causes the loss of electrical conductivity. The second option was to provide a certain time for hydration of the cement, which would allow for the generation of enough portlandite. Then, the pozzolan was added, in order to have a record of the electrical conductivity loss less influenced by the salt dissolution of the cement.
- Temperature and time trial. Maximilien *et al.* [12] used 25 °C and 30,000 s for their experiences. In this study, they did not want the test to be too long, and limited it to 10,000 s reported for calcium hydroxide suspensions [13].

**Table 1:** Main characteristics of previous methods of electrical conductivity measurements in aqueous suspensions.

Reference	Suspension Characteristics	Mineral Admixtures	Assay Times
Sinthaworn * et al.* [4]	Calcium hydroxide solution obtained from Portland cement, without the presence of Portland cement hydrates. Temperature = 80 °C	Silica fume	28 h
		Metakaolin	
		Rice husk ash	
		River sand	
Sinthaworn *et al.* [5]	Calcium hydroxide solution obtained from Portland cement, without the presence of Portland cement hydrates. Temperatures = 40 °C, 60 °C and 80 °C	Silica fume	8 h
Lanzón *et al.* [11]	Aqueous cement suspensions	Lightweight aggregates (slight pozzolanic activity)	1 week
Maximilien *et al.* [12]	Aqueous cement suspensions	No mineral admixture	8.3 h

It well known that the pure pozzolanic reaction involves calcium hydroxide, however, in practice, pozzolans are often used by mixing with Portland cement. The pozzolanic reaction using Portland cement as reagent has been assessed. When a mixture of pozzolan/Portland cement is monitored, two parallel reactions take place: hydration of Portland cement and pozzolanic reaction. This second one depends largely on the first one, because the hydration of Portland cement supplies the portlandite needed for the pozzolanic reaction.

Based on the foregoing, it is evident that a rapid method has not been reported based on measuring the electrical conductivity, to characterize the activity of pozzolanic materials in the presence of Portland cement. Neither was it reported the pozzolanic activity of FCC measured in aqueous suspensions of Portland cement. Therefore, the objective of this study was to discuss and establish the conditions for a rapid method based on the measurement of electrical conductivity of aqueous suspensions of Portland cement that could be useful to characterize the pozzolanic activity of mineral admixtures. In this work, a spent catalytic cracking catalyst was used as pozzolan, and to compare their behavior metakaolin (MK), silica fume (SF) and mullite (MU) were employed.

## 2. Results and Discussion

To achieve the above objectives, six different types of experiments were designed. Experiments 1 and 2 were designed to assess the optimal conditions of the suspension temperature and the amount of cement used (Section 2.1). Experiments 3 and 4 were designed to obtain the most appropriate procedure to add the mineral admixture (Section 2.2). Once achieved, the objectives of the Experiments 1–4 and Experiments 5 and 6 were designed to compare the pozzolanic activity of the FCC with other mineral additives (Section 2.3).

### 2.1. Studies on the Suspension Temperature and the Cement Amount

**Figure 1:**
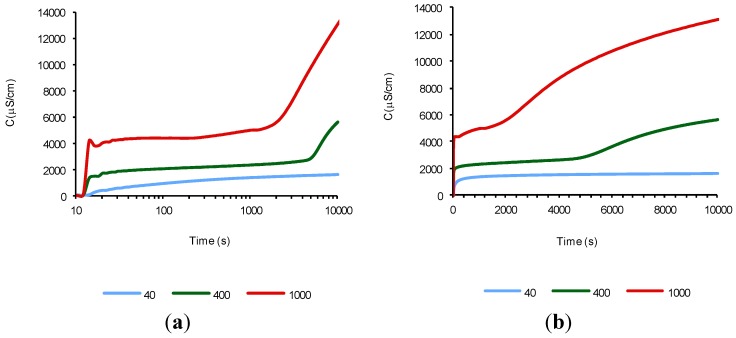
The time evolution of electrical conductivity of aqueous suspensions with several cement amounts (mg), *T* = 40 °C: (**a**) semi logarithmic scale; and (**b**) normal scale.

The objectives of Experiments 1 and 2 were the optimization of the suspension temperature and the cement amount added, while the water volume was maintained constant (in accordance with the conditions of Experiment 1, Section 3.1). The results of the first experiment (50 mL of water at 40 °C for 40, 400 and 1000 mg of cement) are shown in Figure 1. These results are plotted on semi-logarithmic scale (a) and on normal scale (b). The first graph allows us to distinguish more accurately the beginning of the periods of hydration/dissolution, while the second graph allows us to appreciate the finalization of these periods more clearly. It can be appreciated that for the three amounts of cement used, the beginning of the first period (mixing period) is immediate (before 20 s). The ending of this period depends on the cement amount used. As the cement amount present decreases, the ending of this period increases, that is, it takes more time to reach the supersaturation of ettringite and calcium silicates hydrates (CSH) [12]. The second period begins at the end of the previous period, it continues throughout the assay time, and its completion cannot be seen in this experiment. However, in Figure 1b we can appreciate that when the amount of cement increases, the finalization of this period is reached earlier. This conclusion was reached because the decrease in the instantaneous slope of the curve. This slope tends to have a fixed value, from which it can be assumed that the electrical conductivity begins to decrease in the acceleration period. From these facts we can assume that it has not reached the supersaturation of the suspension under the selected conditions of time and temperature. 

**Figure 2:**
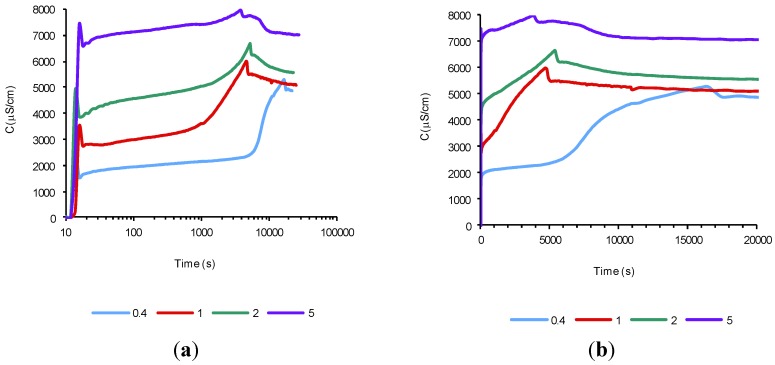
The time evolution of electrical conductivity of aqueous suspensions with several cement amounts (g), *T* = 80 °C: (**a**) semi logarithmic scale; and (**b**) normal scale.

To accelerate the process of portlandite generation and thus reach the end of the second period, it was decided to raise the temperature of the assay to 80 °C (Experiment 2), using the following cement amounts: 0.4, 1, 2, and 5 g. Figure 2 shows the evolution over time of these suspensions on the two scales (semi logarithmic and normal). In these figures, the three periods are clearly distinguishable. It can be observed the end of the induction period (second period) and therefore the beginning of the acceleration period (third period). As previously mentioned, by increasing the amount of cement present (lower *a*/*c* ratio) it can be observed the start of the third period at shorter times. With these results, it was established that it was sufficient to add 1 gram of cement to keep test time at 10,000 s, and thus ensure the portlandite supersaturation in the solution, corresponding to the third period, provided that the suspension temperature was kept at 80 °C.

### 2.2. Optimization of the Way to Add the Mineral Admixture

**Figure 3:**
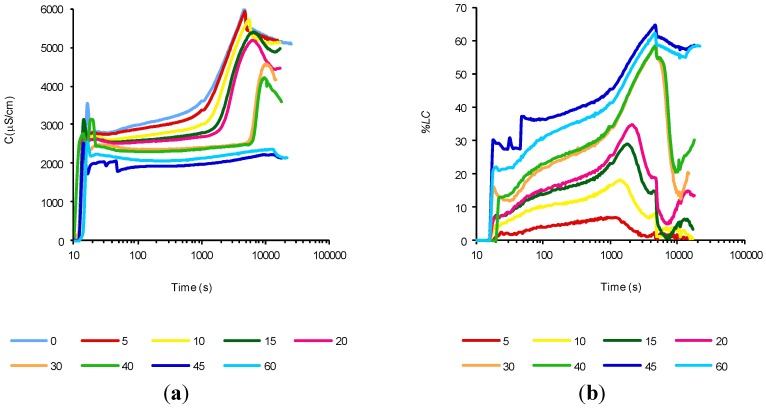
Time evolution of: (**a**) electrical conductivity; and (**b**) loss of electrical conductivity, of aqueous cement suspensions (1 g of cement) with several simultaneous percentages of FCC addition. *T* = 80 °C.

As was mentioned, the purpose of Experiments 3 and 4 was to establish the most suitable procedure to add the mineral admixture to the cement aqueous suspension (see Section 3.2). It was decided not to replace cement by pozzolan in order to maintain the same amount of calcium hydroxide produced by the hydration of cement. Instead of the replacing of cement, an amount of pozzolan was added, while the initial amount of cement was maintained constant. Thus, the aim of Experiment 3 was to study what happens when the pozzolan and the cement are added simultaneously. Figure 3a shows the evolution of the electrical conductivity of 1 gram of cement suspensions added with FCC from 0% to 60% and tested at 80 °C. Figure 3b shows the evolution of the percentage of loss of conductivity (*%LC*) evaluated by the subtraction of the electrical conductivity of the cement/pozzolan suspension from the electrical conductivity of the control suspension (without any mineral addition) at the same time reaction. This allows us to have a *%LC* equivalent that was used in aqueous suspensions pozzolan/calcium hydroxide [13]. This modification is necessary because the cement is continuously hydrating, and therefore the amount of available calcium hydroxide constantly changes and consequently the electrical conductivity. The equation used was:

(1) $$\%LC_{t,c}=\frac{C_{t,c}-C_{t,a}}{C_{t,a}}\cdot 100$$

where,

- *%LC_t,c_*: loss of electrical conductivity of the cement/pozzolan suspension at a given time “*t*”, in percentage terms.
- *C_t,c_*: electrical conductivity of the control suspension at a given time “*t*”.
- *C_t,a_*: electrical conductivity of the suspension with a percentage of pozzolan added at a given time “*t*”.

Table 2 tabulates the values of *%LC* to 100, 1000 and 10,000 s, and the maximum *%LC* value, and the time at which it occurs. Both Figure 3 and Table 2 show that by increasing the amount of catalyst added, the *%LC* increases, reaching a maximum with a 45% addition and slightly decreases with a 60% addition. We can assume that this is because when we add more than 45% of the pozzolan, mixing problems exist and therefore prevents adequate contact between the catalyst and calcium hydroxide reacting appropriately. In Figure 3a also we can observe how as we increase the addition of FCC, the end of the second stage (induction) result in longer time periods. Any completion of this stage depends on the calcium hydroxide supersaturation of the suspension, and since the FCC catalyst continuously consumes the calcium hydroxide, it seems that is not possible to achieve the supersaturation. However, a maximum of *%LC* is reached at a time that seems to have a limit at 4700 s. From this maximum value, all suspensions decrease their conductivity including the control suspension (without any addition), so also decreases the *%LC*. However, at the end of the test, we can still observe a further rise, probably due to an unsaturation, since the pozzolan still consumes the calcium hydroxide.

**Table 2:** Loss of electrical conductivity at 100, 1000 and 10,000 s, and the maximum value of *%LC*, where its time of occurrence is indicated, for cement/water suspensions (1 g/50 mL) with several percentages of FCC addition. *T* = 80 °C.

% FCC (Addition)	*%LC*			
	Time (s)			
	100	1,000	10,000	*%LC*_max_-t_max_ (s)
5	4.00	6.91	1.69	7.06-900
10	10.00	16.57	3.58	18.21-1,400
15	13.67	22.93	4.71	28.81-1,800
20	15.00	25.69	10.92	34.96-2,100
30	21.67	33.70	14.31	58.40-4,700
40	23.00	34.25	21.09	58.57-4,700
45	36.33	45.30	58.00	64.56-4,700
60	30.33	41.16	55.74	62.06-4,700

**Figure 4:**
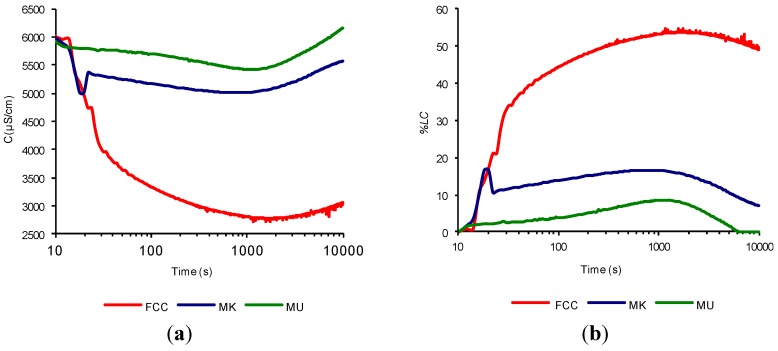
Time evolution of: (**a**) electrical conductivity; and (**b**) loss of electrical conductivity, of aqueous cement suspensions with non-simultaneous addition of FCC, MK and MU.

In order to compare what happens if first the cement is hydrated and then the pozzolan is added, Experiment 4 was performed with a modified procedure (see Section 3.2). The main modifications of Experiment 4 were to decrease the amount of cement (400 mg), the non-simultaneous addition of cement and mineral admixture, and the decrease in temperature of the suspension (40 °C). This type of experiment was conducted for the spent FCC catalyst, metakaolin and mullite (used as non-reactive mineral addition as inert material for comparison), with the addition of 1 gram of these materials. Figure 4a shows the evolution of electrical conductivity over time, while Figure 4b shows the evolution of *%LC* over time. This loss was calculated as reported for aqueous suspensions of pozzolan/calcium hydroxide [13]. This is because the pozzolan was added at 11,000 s, so that the electric conductivity value at that moment can be taken as the maximum value (see Equation (2)). Note that the figures have been plotted starting from the addition of the pozzolan:

(2) $${\left(\%LC\right)}_{t}=\frac{C_{0}-{\left(C_{xa}\right)}_{t}}{C_{0}}\cdot 100$$

where

- (*%LC*)*_t_*: loss of electrical conductivity in the suspension at a given time “*t*”, in percentage terms.
- *C_o_*: electrical conductivity of the suspension before adding the pozzolanic material.
- (*C_xa_*)*_t_* = (*C_xl_*)*_t_* − (*C_x_*): corrected value of the electrical conductivity of the pozzolan/calcium hydroxide suspension at a given time “*t*”, where (*C_xl_*)*_t_* is the electrical conductivity of the pozzolan/calcium hydroxide suspension at a given time “*t*”; and (*C_x_*) is the electrical conductivity of the aqueous suspension of the pozzolan in the absence of calcium hydroxide at the same given time “*t*”.

In previous work [13], it was reported that no correction is necessary for the electrical conductivity due to the salt amount of the pozzolanic material used (FCC, MK and MU), therefore (*C_xa_*)*_t_* ≈ (*C_xl_*)*_t_*. In Figure 4b we can appreciate the huge difference in reactivity of the catalyst FCC, when compared to the MK and the MU, reached a maximum value of *%LC* of 55%, while the MK hardly reaches 16%. After this maximum value, the three pozzolans decrease their *%LC* due to the beginning of the acceleration period previously mentioned. It should be noted that the fourth experimental procedure does not seem to offer more advantages than the previous experiment. This follows because although a larger proportion of catalyst was added, the maximum values of *%LC* obtained in the simultaneous addition were not reached (obviously, the lowering of the temperature to 40 °C was a factor). Besides, the simultaneous addition of the pozzolan and the cement, allows less test time.

### 2.3. Evaluation of Pozzolanic Activity with Optimized Method

The last two experiments were intended to evaluate the pozzolanic activity of the FCC, and comparing it with MK and MU (Experiment 5) and with different silica fumes (Experiment 6). The optimum conditions for the method are established as (see Section 3.3):

- Simultaneous addition of cement and pozzolan.
- Suspension temperature of 80 °C.
- 1 g of cement in 50 mL of water.

**Figure 5:**
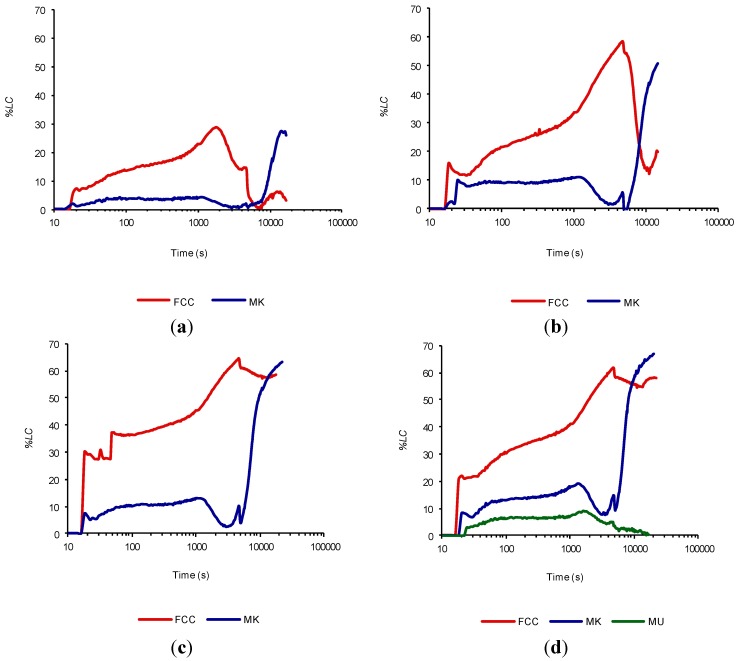
Time evolution of the loss of electrical conductivity of cement/water suspensions (1 g/50 mL) at 80 °C with the following percentages of addition for FCC and MK: (**a**) 15%; (**b**) 30%; (**c**) 45%; and (**d**) 60% (MU for comparison).

Figure 5 shows the curves for the evolution of the loss of electrical conductivity calculated according to Equation (1) for the catalyst and metakaolin in the following addition values: 15%, 30%, 45% and 60%. Mullite was evaluated only with a 60% of addition due to its low reactivity. For all the percentages of the addition we observe that the catalyst is much more reactive than metakaolin. It seems that at the end of the trial, the MK begins a strong pozzolanic activity, confirming that the FCC reacts much earlier than MK, as has been reported in thermogravimetric studies [14]. It is also clearly seen that when the addition of FCC increases the *%LC* also augments, as was mentioned in the previous section.

This behavior can be explained as follows: the FCC is very reactive and begins to react as calcium hydroxide is released (*%LC* increases). In this way, a maximum value of *%LC* is reached, when there is no longer enough calcium hydroxide to continue reacting. The cement hydration continues with the generation of portlandite, which reduces the *%LC*. On finalizing the test when the amount of available calcium hydroxide increases due to continuous hydration of the cement, the FCC reacts again. MK requires a greater amount of calcium hydroxide to initiate the reaction, so that its reaction does not start until the suspension is completely supersaturated in calcium hydroxide.

We can consider the area under the curve of *%LC* as the “work” to keep the loss of electrical conductivity to a given value (since cement is continuously hydrating and producing portlandite). We can also see in Figure 5 how this area increases significantly as the percentage of added catalyst also increases. To quantitatively confirm this data, the area was calculated in the range from 10 to 10,000 s. For this calculation we consider the width of the rectangles at their base as: 2 s in the range of 10 to 100 s, 10 s in the range of 100 to 1000 s, and 100 s in the range of 1000 to 10,000 s. The height of the rectangles is determined from the curve of *%LC*. Finally, the total area is obtained by summation of all individual areas of the rectangles for each curve. Table 3 tabulates the values of these areas. We appreciate how the area under the curve increases by increasing the percentage of the addition of the pozzolan. The FCC reaches a maximum area value with the 45% addition, since this value is almost constant when passing from 45% to 60% addition of FCC. This behavior may probably be due to depletion of calcium hydroxide produced by the hydration of cement (and consumed by the FCC). It is remarkable that the FCC area is approximately three times the MK area to the same percentage of the addition (in 15%, 30% and 45% addition), and twice for the addition of 60%. From these studies we can conclude that the pozzolanic activity of the catalyst is much greater than MK and the MU for the test conditions established. Perhaps if we continue the test for a longer period of time, we should observe the strong pozzolanic activity of MK.

**Table 3:** Calculated areas under the curve of *%LC* from 10 to 10,000 s for cement suspensions.

Addition (%)	Pozzolan	Area (%·s) ×10^5^
15	FCC	10.75
	MK	3.16
30	FCC	38.43
	MK	11.80
45	FCC	56.94
	MK	18.69
60	FCC	54.11
	MK	25.97
	MU	4.12

**Figure 6:**
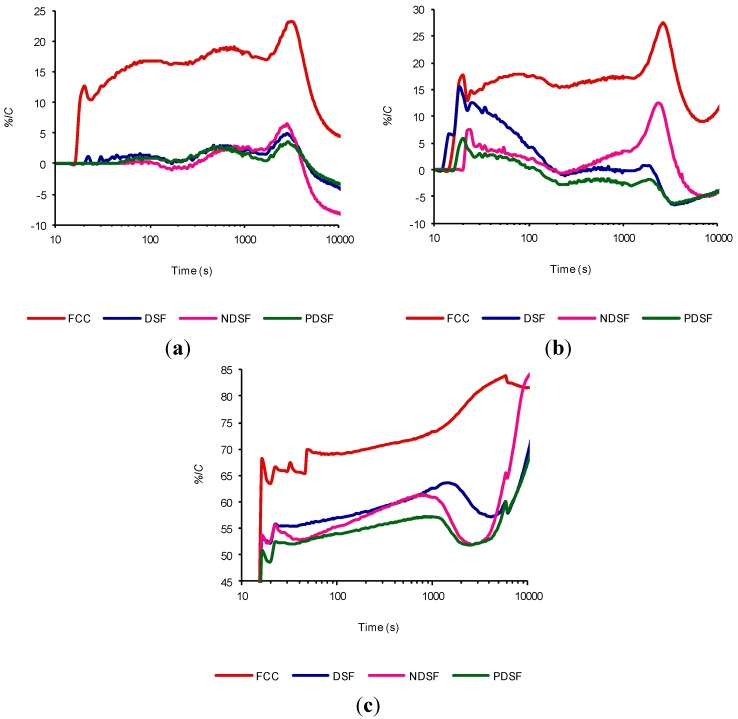
Time evolution of the loss of electrical conductivity of cement/water suspensions (1 g/50 mL) with 45% of addition of FCC and SF at the following temperatures: (**a**) 30°C; (**b**) 40°C; and (**c**) 80 °C.

Having established the assay conditions and catalyst performance compared with metakaolin, finally we also wanted to compare the catalyst with several silica fumes in the same conditions (see Section 3.3, experiment 6). Figure 6 shows the evolution over time of the loss of electrical conductivity *%LC* calculated from the Equation (1) for these suspensions. We can make the following observations:

- For the three tested temperatures, the *%LC* of the catalyst is much higher than in the three silica fumes in the established conditions.
- In general based on the *%LC*, the pozzolanic reactivity of silica fumes tested high to low is: NDSF > DSF > PDSF, although there are areas where this pattern is lost. The PDSF contradictory behavior may be because it is obtained from a different source.
- The negative values for the silica fumes should be because they are contributing to accelerating the cement hydration. This causes a reading of an instantaneous electrical conductivity higher than that recorded in the control suspension, thus causing negative values. These negative values are mainly presented after reaching the maximum value of *%LC*.
- Considering the area under the curve as the “work” of the pozzolan to keep the *%LC* at a certain value, we see that the FCC develops more “work” than the silica fumes during the time interval tested. However, as happens for MK, it appears that if given a longer test time, the NDSF especially would show an important pozzolanic activity (especially at 80 °C, where at the end of trial, the *%LC* value of silica fume are equalized with the FCC value).

By increasing the temperature of the suspension, the start of the third period seems to shift slightly to longer times caused by the competition between two processes. On the one hand, the greater generation of portlandite tends to end the induction period, and on the other hand the pozzolanic reaction is accelerated by temperature and decreases the portlandite concentration of the suspension, causing a delay in the completion of this period. Notably we observed that for the spent FCC catalyst, the dominant process is the pozzolanic reaction (since there is a shift to longer times on the completion of the second period), while for the silica fumes the opposite occurs, indicating that the pozzolanic reaction is subordinated to the hydration of cement (probably accelerated by the silica fume, as was aforementioned).

## 3. Experimental Section

In preparation for the experiments, FCC was ground for 20 min in an alumina ball mill to increase its reactivity [15]. This drastically reduces particle size from 73.94 to 19.95 µm, and obtaining a Blaine fineness of 11,530 cm^2^/g. This catalyst was supplied by British Petroleum (BP OIL) Spain S.A. (Castellón, Spain). To compare the pozzolanic behavior of the catalyst, Metacaolin (MK, supplied by ECC International Company (St Austell, UK), under the trade name Metastar) was used as a pozzolanic material, which has a chemical composition very similar to FCC. Silica fume was also used for comparative purposes, since it is a widely used high reactivity pozzolanic material. Three kinds of silica fume were used: non-densified silica fume (NDSF) and densified silica fume (DSF), both supplied by FerroAtlántica SA (Sabón–A Coruña, Spain) and partially densified silica fume (PDSF) supplied by SIKA (Gournay-en-Bray, France). Ordinary Portland cement (OPC) was used (Cemex, Valencia, Spain, Blaine fineness of 3968 cm^2^/g). The chemical compositions of the OPC, FCC, MK and SF are tabulated in Table 4. An inert crystalline material was used, mullite (MU, Al_6_Si_2_O_13_), in order to establish a comparison of the pozzolanic behavior of the FCC. The chemical composition of the MU, since is similar to the FCC allows us to study the effect of dilution. The percentages of aluminum and silicon oxides in the MU are respectively 71.8% and 28.2%. The average particle diameters for Portland cement and mineral admixtures studied are: cement = 15.01 µm, FCC = 19.96 µm, MK = 5.84 µm, NDSF = 8.81 µm, DSF = 44.41 µm, PDSF = 29.01 µm, MU = 37.36 µm. The equipment used for measuring the electrical conductivity is as described by Paya *et al.* [2]. This equipment uses a microCM2201 Crison conductivity meter (Alella, Spain) with a RS232 output. The experiments were carried out through a thermostated reactor for temperature control as well as by using an isolated system to avoid carbonation. For optimization of the experimental conditions of the assessment of the pozzolanic activity the measurements of the loss of electrical conductivity, four kinds of experiments were designed.

**Table 4:** Chemical composition (% in weight).

Material	SiO_2_	Al_2_O_3_	Fe_2_O_3_	CaO	MgO	SO_3_	K_2_O	Na_2_O	LOI
OPC	19.9	5.38	3.62	63.69	2.14	3.66	1.17	0.10	2.02
FCC	48.2	46.0	0.95	<0.01	<0.01	n.d. ^§^	<0.01	0.50	1.50
MK	52.1	41.0	4.32	0.07	0.19	n.d. ^§^	0.63	0.26	0.60
DSF–NDSF	91.1	0.2	0.14	0.48	0.19	0.14	0.50	n.d. ^§^	6.69
PDSF	96.0	0.2	0.14	0.36	0.20	0.12	0.41	0.02	2.94

^§^ Not determined.

### 3.1. Experiments 1 and 2

In these experiments, the objective was to establish the optimal conditions of suspension temperature and the amount of cement used. In both experiments 50 mL of water were used. In the first experiment the suspension temperature was set at 40 °C, by varying the amount of cement used, these being 40, 400 and 1000 mg. In the second experiment the suspension temperature was 80 °C, by varying the amount of cement added in the following values: 0.4, 1, 2, and 5 g. In these two first experiments not mineral admixtures were used.

### 3.2. Experiments 3 and 4

In these experiments, the aim was to establish the optimal way to add the mineral admixture. In both experiments, the water volume was 50 mL. It was decided to make an addition to the cement amount, not a substitution. Therefore in the third experiment the conditions were: Percentages of pozzolan added with respect to the mass of cement (only FCC) 0%, 5%, 10%, 15%, 20%, 30%, 40%, 45% and 60%, 1 g of cement amount, suspension temperature 80 °C. The cement and pozzolan were added simultaneously. In order to compare what happens if we first allow the cement hydration and then the pozzolan is added, a fourth experiment was conducted using the following procedure: (1) 400 mg of cement was added to 50 mL of water at a temperature of 80 °C for 1000 s; (2) the temperature was decreased to 40 °C and maintaining the system at 10,000 s; (3) one gram of the pozzolan was added. This study was conducted for the FCC, MK and MU.

### 3.3. Experiments 5 and 6

Once optimized the experimental conditions of the assessment of the pozzolanic activity by the measurement of the loss of electrical conductivity, then two other types of experiments were designed in order to compare the FCC pozzolanic activity with other mineral additives. The optimized conditions of these two experiments were: 80 °C of the aqueous suspension temperature, 1 g of cement in 50 mL of water, with the simultaneous addition of cement and the mineral addition. In experiment 5, FCC was compared with MK with the following addition percentages: 15%, 30%, 45% and 60%. MU was compared only with 60% addition. In Experiment 6, the FCC was compared with three types of SF. This comparison was made with an addition of 45% of the pozzolan, at three different suspension temperatures: 30, 40 and 80 °C.

## 4. Conclusions

From the present study of the pozzolanic activity measured by the electrical conductivity of cement aqueous suspensions, the following conclusions can be drawn:

1. The assay conditions were optimized for measuring the pozzolanic activity by means of the electrical conductivity in cement aqueous suspensions. It was established as optimal conditions for the spent FCC catalyst: test time = 10,000 s; suspension temperature = 80 °C, water/cement ratio = 0.50; simultaneous addition of cement and pozzolan.
2. Applying the method to the suspensions allowed us to observe the three periods described by Maximillien [12] in which significant changes occurred in electrical conductivity: mixing, induction and acceleration.
3. In the test where the initial hydration of the cement in the aqueous suspension is allowed, a large difference in reactivity is obtained when comparing the FCC, MK and MU. The FCC shows the *%LC* maximum value of 55, while MK barely reaches to 16%. This method does not seem to provide advantages over the simultaneous addition, since although a greater proportion of spent FCC catalyst was added, the maximum values in *%LC* obtained in the simultaneous addition were not reached, and a larger test time is required.
4. When comparing FCC with MK in all experiments performed, it was observed that for the given conditions, the spent FCC catalyst is more reactive than metakaolin. Besides, it is evident that the increase of FCC addition increases the *%LC*.
5. When comparing the catalyst with three types of silica fume in cement aqueous suspensions in the three tested temperatures, the *%LC* of the spent FCC catalyst is far superior to established test conditions. In general based on the *%LC*, the pozzolanic reactivity of the silica fumes tested from high to low is NDSF > DSF > PDSF. Negative values obtained for the silica fumes are because they contribute to the acceleration of cement hydration.
